# Headache and Associated Psychological Variables in Intensive Care Unit Nurses during the COVID-19 Pandemic: A Prospective Study

**DOI:** 10.3390/jcm13133767

**Published:** 2024-06-27

**Authors:** Fernanda Gil-Almagro, Francisco Javier Carmona-Monge, Fernando José García-Hedrera, Cecilia Peñacoba-Puente

**Affiliations:** 1 Faculty of Health Sciences, Department of Psychology, Universidad Rey Juan Carlos, Av. de Atenas, s/n, 28922 Alcorcón, Madrid, Spain; fgilalmagro@gmail.com; 2 Nurse Intensive Care Unit, Hospital Universitario Fundación Alcorcón, Budapest, 1, 28922 Alcorcón, Madrid, Spain; fjgarciah@gmail.com; 3 Anesthesia Department, Hospital Universitario Santiago de Compostela, Rúa da Choupana, s/n, 15706 Santiago de Compostela, A Coruña, Spain; javichun@gmail.com

**Keywords:** headaches, stress, cognitive fusion, hardiness, nurse, intensive care

## Abstract

**(1) Background:** Headaches in health professionals have been studied over the years. This has become even more relevant during the COVID-19 pandemic, due to their link with the use of masks, being female or working in highly complex units. However, their association with different personality traits has not been studied in healthcare workers (HCWs). The aim of this study was to assess the prevalence and evolution of headaches throughout the pandemic in Intensive Care Unit (ICU) nurses and to analyze their association with sociodemographic, occupational and personality variables as well as with other symptoms. **(2) Methods:** This was an observational, descriptive, longitudinal, prospective study with two periods of data collection (at the end of the containment phase and six months thereafter). A non-probabilistic convenience sampling was performed (n = 131). **(3) Results:** A high percentage of ICU nurses reported headaches during the first (71%) or second (79.4%) time point; more than half of the sample (58.8%) reported headaches over time (chronic headache). Although a higher prevalence of headaches was observed in women at both assessment points, no significant gender-related relationships were observed for headaches maintained across the two time points. Regarding the symptoms and personality variables, positive relationships were found between headaches and anxiety (*p* = 0.005), insomnia (*p* = 0.030) and emotional exhaustion (*p* = 0.022), and a negative relationship was found between headaches and hardiness (*p* = 0.031). **(4) Conclusions:** Our study highlights the importance of assessing occupational, psychological and emotional aspects in order to decrease the prevalence of headaches and thus improve the quality of the work life of nurses in such demanding environments as the ICU.

## 1. Introduction

Headaches are a highly prevalent symptom in the general population, especially among females, and have been associated with tension processes, insomnia and occupational stressors [1]. In the context of the COVID-19 pandemic, as a situation of extreme stress among healthcare workers (HCWs), emotional symptoms have been extensively studied [2], though research on physical symptoms in these professionals has been considerably less. 

Within headaches, the previous literature indicates different types in the general population, including migraine, tension headache, cluster headache, various headaches not associated with structural injury, headache associated with head trauma, headache associated with vascular disorders, headache associated with non-vascular intracranial disorder, headache associated with substances or their withdrawal, headache associated with non-cephalic infection, headache associated with metabolic disorder, headache or facial pain associated with disorders of the skull, neck, eyes, ears, nose, sinuses, teeth, mouth or other facial or cranial structures, cranial neuralgia, nerve trunk pain and unclassifiable headache [3]. However, according to health professionals, and despite the fact that headaches are considered a symptom with high prevalence in this group [4], there are few studies that classify the different types of headaches within HCWs. Scientific evidence has been found regarding nurses, referring to a classification that divides headaches into frequent headache, migraine and tension-type headache [5]. 

The high frequency of headaches among HCWs was highlighted even before the COVID-19 pandemic [6], with percentages of up to 88% and with a clear predominance among females [6]. This high rate of headaches in these professionals is associated with the high levels of stress to which they are subjected. Perceived stress has been shown to increase muscle activity, which directly contributes to headache formation [7]. Several studies have shown a higher prevalence of headaches in nurses than in the general population [8]. Specifically, these studies have shown that an increase in workload is associated with an increase in the prevalence of headaches, mental fatigue and neck pain [9]. Likewise, a relationship between shift work and headaches has been observed [10,11], and working in highly specialized care services such as the Intensive Care Unit (ICU) has been suggested to be an additional risk factor [12,13].

Given that headaches have been associated with COVID-19 symptoms, in the specific context of the pandemic, the possible risk factors for headaches in HCWs have been analyzed, with special interest in working conditions and vaccination [14,15,16,17,18,19]. A direct relationship has been found between an increase in headaches [14,15] and the use of personal protective equipment (PPE), in particular the use of masks, with percentages of up to 70% [16,17], coining the term “facemask headache”. Furthermore, an association between the COVID-19 vaccine and headaches has also been described, with up to 30% of HCWs reporting post-vaccination headaches. This association is even more pronounced among women [18,19]. 

The study of headaches in healthcare personnel during the COVID-19 pandemic has been carried out mainly by means of correlational observational designs, analyzing headaches’ relationship with certain working conditions [8,9,10,11,12,13]. To the best of our knowledge, there is no research analyzing the association between headaches and psycho-emotional symptoms beyond the stress experienced during the pandemic, or regarding their relationship with other types of highly prevalent symptoms among HCWs during the COVID-19 pandemic such as insomnia or burnout. The study of personality variables that may be associated with the prevalence and chronification of headaches in HCWs is a topic of special interest in health promotion, highlighting the impact of certain psychological variables (as protective or risk factors) on the mental health of healthcare personnel during the COVID-19 pandemic, including traits such as cognitive fusion [20] or hardiness [21]. However, no research in relation to headaches has been carried out.

In an attempt to provide information in this regard, the purpose of this research was to study the prevalence and clinical features of headaches among intensive care unit nurses during the COVID-19 pandemic and to determine the association of headaches with sociodemographic, occupational, psychiatric and personality factors.

Based on the above-mentioned observations, we hypothesize high prevalences of headache in ICU nurses, around 70%, at the beginning of and six months after the COVID-19 pandemic. A positive relationship between headache and anxiety, insomnia and burnout is also hypothesized as an additional hypothesis. Finally, we expect to find a negative association between headache and cognitive fusion and a positive association between headache and hardiness.

## 2. Materials and Methods

### 2.1. Design

This was an observational, descriptive, longitudinal, prospective study with two data collection periods: (1) between 5 May and 21 June 2020 (final phase of the state of alarm in Spain) and (2) a follow-up 6 months later (January–April 2021). At both time points, the presence of headaches was assessed, as were 12 additional COVID-19 symptoms. 

**Figure 1:**
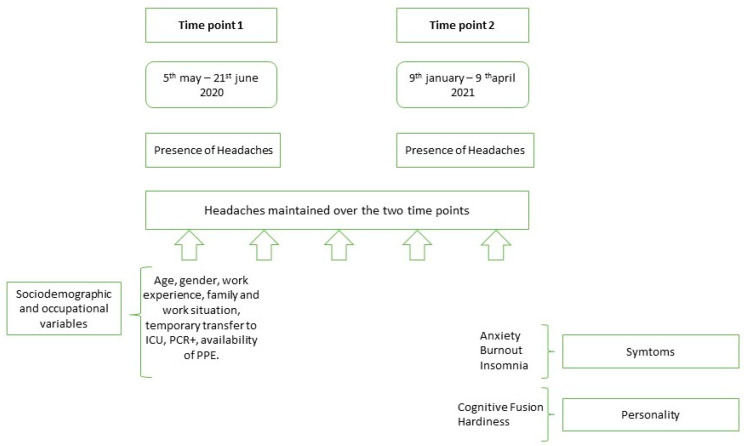
Assessment time points and variables involved in the study.

In order to achieve the research aims, different sociodemographic, occupational and personality variables were evaluated, as were symptoms. Thus, at the first time point, certain sociodemographic and occupational data were evaluated, such as age, gender, marital status, years of experience in the unit, work situation and work shifts. At the second time point, symptoms were assessed, specifically anxiety, insomnia and burnout, as well as personality variables such as cognitive fusion and hardiness (see Figure 1; see section on instruments for more detailed information).

### 2.2. Population and Sampling

The sample consisted of 131 ICU nurses. The sample was selected by non-probabilistic convenience sampling. Currently, in Spain, there is no registry of nurses working in the ICU, although there is a society called the Spanish Society of Intensive Care Nurses and Coronary Units (Sociedad Española de Enfermería Intensiva y Unidades Coronarias -SEEIUC-), which currently has 815 registered critical care nurses. In this population, the calculated sample was 68 for a 95% confidence interval. Recognizing that the number of nurses registered in the society (SEEIUC) would be lower than the number of nurses working in intensive care units, the sample size was selected on a methodological basis. Specifically, a minimum number of n = 120 was considered as the sample size for prospective studies [22]. The following inclusion criteria were taken into account: working as an ICU nurse during the data collection period and being in direct contact with COVID-19 patients. Exclusion criteria included a change of service during the period of data collection and working as a nurse manager. Aware of the usual sample loss in longitudinal studies with this population, and also taking into account the added contextual difficulty of data collection (COVID-19 pandemic) [23,24], a minimum sample size of 300 participants was established at the first time point, finally resulting in a sample of 334. Of these, at the second time point 6 months later, 131 nurses continued their participation, constituting the final sample of the present investigation. 

The evaluation was carried out by means of a self-report protocol that included both an ad hoc questionnaire designed by the research team with the sociodemographic and occupational variables and the COVID-19 symptoms, including headache, and the rest of the standardized questionnaires that allow for the evaluation of the variables of interest in this study. The objective of the study was presented at the beginning of the assessment protocol, and the informed consent of the participants was requested, in particular the use of e-mails to establish contact during the evaluation periods of the research. The study was circulated by sending the link to the questionnaire through social networks (Facebook, Twitter and WhatsApp) and through corporate e-mails of the public and private health services of the Spanish system. 

In accordance with the prospective research design previously discussed, two evaluation periods were carried out, the first of which took place between May and June 2020 [time point 1]. The e-mails of the nurses who had participated in that period were used to carry out the second evaluation period, which took place between January and April 2021 [time point 2]. 

### 2.3. Variables and Instruments

#### 2.3.1. Presence of Headache [Time Point 1 and 2]

Headaches were assessed within the set of symptoms associated with COVID-19. In particular, a self-report questionnaire developed ad hoc by the research team was used. The questionnaire included a total of 13 symptoms. In addition to headache, these included fever, chills, cough, myalgia, respiratory distress, dizziness, rhinitis, sore throat, chest pain, anosmia, ageusia and skin manifestations. The questionnaire was headed with the question, “Have you experienced any of the following COVID-19-susceptible physical symptoms during the past few weeks?”. Each of the symptoms (including headache) presented a yes/no response format.

#### 2.3.2. Sociodemographic and Occupational Variables [Time Point 1]

Sociodemographic variables (age, gender, marital status) and occupational variables (years of service experience and stability in employment) were collected.

#### 2.3.3. Variables Related to Symptoms [Time Point 2]

(a) Anxiety

The Spanish version of the Generalized Anxiety Disorder Scale (GAD-7) was used [25] to assess the existence of symptoms compatible with generalized anxiety disorder. The scale is composed of 7 items in a Likert format with 4 response options, ranging from 0 (not at all) to 3 (almost every day), so that higher scores on the scale indicate greater severity of symptoms. The internal consistency in the present study was 0.93.

(b) Insomnia

The Insomnia Severity Scale (ISI) [26] in its Spanish version [27] was the instrument used for the evaluation of insomnia. It is a questionnaire that measures insomnia briefly, following the criteria of the Diagnostic and Statistical Manual of Mental Disorders and the International Classification of Sleep Disorders. It is composed of 7 items that provide information on three dimensions (severity, impact and satisfaction). Each item is answered on a Likert-type scale from 0 (no problem) to 4 (many problems), obtaining a total score between 0 and 28, establishing the cut-off point at 22 for severe clinical insomnia. Cronbach’s alpha for this scale in the present study was 0.87.

(c) Burnout

The Maslach Burnout Inventory-Human Services Survey (MBI-HSS) was used [28] in its Spanish version [29]. It consists of 22 items that adopt a 7-point Likert-type response format, ranging from 0 (never) to 6 (every day). The instrument assesses three dimensions or subscales of burnout: emotional exhaustion, depersonalization and reduced personal accomplishment. In our study, Cronbach’s α was 0.88 for the full scale. Regarding the subscales, Cronbach’s α was 0.90 for emotional exhaustion, 0.72 for depersonalization and 0.84 for personal accomplishment.

#### 2.3.4. Personality-Related Variables [Time Point 2]

(a) Cognitive Fusion

The Cognitive Fusion Questionnaire (CFQ) was used [30] in its Spanish version [31], composed of 7 items assessing cognitive fusion (the extent to which we are psychologically entangled with or dominated by the form and content of our own thoughts). All items are answered on a 7-point Likert-type response scale, ranging from 1 (never) to 7 (always). Cronbach’s alpha was 0.97 in our study.

(b) Hardiness

The Occupational Hardiness Questionnaire (OHQ) was administered in its Spanish version [32]. It is composed of 17 items that assess the three dimensions of hardiness (commitment, control and challenge), using a Likert-type response format ranging from 1 (“completely disagree”) to 4 (“completely agree”). For the present study, the total hardiness personality score was considered, so that the higher the score, the higher the hardiness personality [33]. Cronbach’s alpha in our study was 0.82.

### 2.4. Data Analysis

Statistical analysis of the data was performed with IBM SPSS Statistics version 27.0 software (IBM Corp. Armonk, New York, NY, USA). Descriptive data such as means, standard deviations, frequencies and percentages were used. Analyses of the associations between variables were performed with Pearson’s correlations, χ2 tests, Student’s *t* tests or one-factor ANOVAs, depending on the type of variable. Cronbach’s α were calculated. Statistical significance was estimated for values of *p* < 0.05.

## 3. Results

### 3.1. Characteristics of the Sample

Table 1 shows the sociodemographic and occupational characteristics of the participants. As can be seen, a total of 131 ICU nurses finally participated with a mean age of 40.54 (SD = 10.02; range 22–60). Of the sample, 88.5% (n = 116) were women, and the majority were married or cohabiting with a partner (67.2%). Regarding work variables, the years of experience in the unit ranged from no experience (newcomers to the unit) to 35 years of experience (mean = 11.76; SD = 9.34). Specifically, 22% had been transferred to the ICU during the COVID-19 pandemic. Most of the nurses were permanent (59.5%), with the most frequent type of shifts being rotating (43.1%) and more than 10 h (36.2%).

### 3.2. Prevalence of Headaches and Their Evolution over Time (Chronic Headaches)

A total of 71% of the sample (n = 93) reported headaches at the first time point, and 79.4% (n = 104) reported headaches at the second time point. Of the total sample, 58.8% (n = 77) reported headaches at both time points at which the assessment was performed (chronic headaches). Headaches subsided for only 12.2% (n = 16) at the second time point (compared with presence at the first time point).

### 3.3. Relationship of Headaches with Sociodemographic and Occupational Variables

Table 1 shows the results of the associations between headaches and the sociodemographic and occupational variables considered, taking into account both the presence of headaches at the first moment of evaluation (end of the confinement phase) and their chronification (maintenance six months later). As can be seen in this table, statistically significant differences are only found for gender (*p* = 0.036) regarding the presence of headaches at the first time point (at the end of the confinement period), with an effect size of 3.27. Specifically, in the case of women, 74% reported experiencing headaches, whereas men were split evenly (around 50%). These differences were diluted when chronic headaches were assessed; the differences in the percentages between men and women became non-significant (*p* = 0.311).

When analyzing the work situation (permanent vs. non-permanent), significant tendencies (*p* = 0.086) were observed with regards to the presence of headaches at the first time point. Thus, the percentage of nurses reporting headaches was higher (79.2%) among the non-permanent nurses compared to nurses with stable employment status (65.4%). These differences disappeared when chronic headaches were considered (*p* = 0.164).

No statistically significant differences or trends towards significance were observed in the other variables considered: age, years of experience, family situation, work situation, hours of work per week, work transfer to the ICU, positive CRP and availability of PPE.

### 3.4. Associations between Headache Maintenance, Symptoms (Anxiety, Burnout, Insomnia) and Personality (Cognitive Fusion and Hardiness)

#### 3.4.1. Symptoms

Table 2 shows the differences in anxiety, insomnia and burnout syndrome (emotional exhaustion, depersonalization and reduced personal accomplishment) between nurses experiencing headaches throughout the two evaluation periods of the study (chronic headaches) and those who did not. As can be seen in the table, the mean scores for the total sample were consistent with mild anxiety symptoms. Nurses with chronic headaches had significantly (*p* = 0.005) higher anxiety scores (mean = 9.64) than those who did not experience headaches (mean = 7.22). The observed effect size is medium (Cohen’s d = 0.51).

Regarding insomnia, the mean scores for the total sample were consistent with subclinical insomnia. Nurses with headaches during both time points had significantly higher scores (*p* = 0.030) for insomnia than nurses who did not report chronic headache. The effect size is small–medium (Cohen’s d = 0.39).

The three dimensions of burnout (emotional exhaustion, depersonalization and personal accomplishment) were assessed independently. The mean scores for the total sample indicated high emotional exhaustion, medium depersonalization and reduced personal accomplishment. Nurses with chronic headaches showed higher emotional exhaustion than those without headaches (*p* = 0.022), with small–medium effect size (Cohen’s d = 0.41).

#### 3.4.2. Personality

Table 2 also shows the differences in cognitive fusion and hardiness between nurses who presented with chronic headaches and those who did not. In the case of cognitive fusion, significant differences were observed (*p* = 0.049). Specifically, nurses with chronic headaches had higher cognitive fusion scores than those without headaches. Regarding hardiness, nurses with chronic headaches had lower scores on hardiness than those who did not experience headaches, the difference being statistically significant (*p* = 0.031). The observed effect sizes are small in all cases, especially for cognitive fusion.

## 4. Discussion

Headaches are a common symptom among nursing professionals, especially in high-demand settings such as ICUs [34]. Understanding the psychosocial factors associated with headaches in this specific group of healthcare workers is critical to adequately address this problem. In the context of the COVID-19 pandemic, though the association with certain occupational variables (i.e., use of PPE and facemasks) has been extensively studied, the relationship of headaches with certain psychosocial variables has hardly been explored. In view of this need, the current study explores the association between psychosocial variables and headaches in a specific group of health professionals such as ICU nurses.

As far as sociodemographic variables are concerned, our results show an absence of a relationship between age and headaches in ICU nurses. This result is consistent with previous research that has shown an unclear relationship between age and the prevalence of headaches in the general population, with great variability depending on the group studied [35]. Our findings seem to show that in the context of ICU nurses, factors other than age may have a more significant influence on the prevalence of headaches [36].

On the other hand, our study supports the idea that women suffer more headaches than men in this specific work environment. This finding is consistent with the existing literature documenting a higher prevalence of headaches in women than in men [6]. This finding not only applies to hospital settings; it is generalizable to the general population and other occupational settings [37,38]. It has been suggested that this gender disparity may be due to hormonal fluctuations, gender roles and differences in pain perception and expression [39]. It is crucial to consider these differences when designing headache management interventions aimed at ICU nurses. A particularly relevant finding of our results is that gender is no longer a significant variable in relation to headache maintenance over time. That is, though a statistically significant higher prevalence of headache onset in response to an excessively stressful situation was observed in the case of women, when assessing cases of chronic headache (persistent six months later), no differences were observed between men and women. Given that to the best of our knowledge, there are no studies on this subject, it could be suggested that once headaches are established as a response to stress, their maintenance depends on other variables more closely related to cognitive and emotional aspects of pain processing, as has been pointed out in the previous literature [40,41].

Some studies indicate that working in highly specialized services may contribute to increased headaches, and that years of work experience could be a buffer against symptomatology [42]. However, our results found no relationship between years of experience and headache, perhaps because the very stressful characteristics of the pandemic (high impact and uncertainty) did not facilitate the buffering role of experience on adaptive coping in the stressful situation. Other psychosocial aspects such as family and work situation may play a relevant role in the manifestation of headaches. The lack of association between family status (married or single) and headaches is an interesting finding that contrasts with studies that have identified stress derived from marriage as a risk factor for headaches [42,43]. In this respect, it is worth mentioning interactionist models that allude to the need to incorporate contextual variables in order to understand the possible relationships between variables [44]. In relation precisely to these contextual variables, the data collection of this study was carried out during the COVID-19 pandemic. Specifically, the first period of collection was carried out during the confinement phase in Spain, which was characterized by the avoidance of social contact and social support, so that in this time period, the relationship with a partner may have had a special relevant value in stress processes (buffer or risk), as in some cases, this may have been the only source of “physical” social support available.

Similarly, employment status (permanent vs. non-permanent) was not associated with increased headaches in ICU nurses, which may suggest that other work factors may have had a more significant influence on nurses’ health. The evidence suggests that work stress, working night shifts or the use of personal protective equipment against infectious patients may have contributed more specifically to the increase in headaches during the pandemic in nurses [9,17,45]. Special mention should be made here of the lack of significant results between headache and use of PPE, in contrast to previous studies [15,17,46]. A possible explanation for this seemingly contradictory result could be related to the PPE indicator that is actually associated with headache. Thus, previous studies indicate that the risk factor for headache is the lack of PPE–person fit (i.e., ergonomic adaptation) and not the availability of PPE itself [47,48]. Given the absence of PPE during the COVID-19 pandemic, this was a risk factor of particular interest. On the other hand, in order to understand the non-significant PPE-headache results in our research, the wide variability of equipment included in PPE must also be taken into account. Thus, most of the previous research linking PPE and headache focuses on the specific use of facemasks (i.e., facemask headache) [16], and in our study, we evaluated the use of PPE in a generalized way, without specific mention of their types and specifically of facemasks.

Regarding the variables analyzed in our study that refer to symptoms, and according to our hypotheses, we can highlight a significant association between anxiety, insomnia and emotional exhaustion, causing an increase in the prevalence and chronification of headaches in ICU nurses. These results are in line with previous scientific evidence that has identified psychological stress as an important risk factor for chronic headaches [9]. Burnout, and specifically emotional exhaustion, has also been linked in the literature to increased headaches in HCWs [3,9]. Specifically, ICU personnel have been experiencing high levels of burnout for years [49], which leads to an increase in symptoms such as headaches [8]. Thus, effective management of these psychological risk factors may play a key role in the prevention and treatment of headaches in this group of healthcare workers.

One of the most novel findings of the present study, compared to others, is the focus on the association between headaches, and specifically their maintenance, with personality variables. These data are of special interest when compared to the scarce role that sociodemographic and occupational variables, including workload or availability of PPE, have shown in our study in relation to headaches. Even the role of gender, with a higher risk of headaches in the female population having been established in the previous literature, is of particular interest [6,42], as this association appears to be diluted when headaches are assessed at both a single time point at the beginning of the pandemic (acute stress) and also in relation their maintenance six months later. 

Specifically, a variable that has hardly been studied in the context we are dealing with, but which has nevertheless been extensively researched in other fields, [50,51] is cognitive fusion. Our data suggest there is an association (close to significance) between cognitive fusion and an increased risk of headaches in ICU nurses (*p* = 0.051). To the best of our knowledge, cognitive fusion has scarcely been studied in HCWs during the COVID-19 pandemic. The few existing studies indicate, in samples of nurses, that this has a role as a risk factor, in addition to mental fatigue [20]. Furthermore, there is evidence that it also plays a mediating role between work-related stressors and mental health problems [52]. In patients suspected of COVID-19, the mediating role of cognitive fusion between experienced stress and levels of anxiety and depression has been shown [53].

The association between cognitive fusion and headaches found in our study highlights the importance of promoting nursing interventions aimed at identifying, regulating and establishing healthy relationships with thoughts. Cognitive fusion, defined as the tendency to confuse thoughts with reality, may be related to perceived stress and rumination, factors that have been previously associated with headaches [7,9]. In fact, cognitive fusion has been linked to increased stress in HCWs [20]. However, to the best of our knowledge, there are no previous studies associating increases in cognitive fusion with increased prevalence or chronification of headaches, either in the general population or specifically among HCWs.

Regarding hardiness, characterized by traits of commitment, control and challenge in the face of stressful situations, previous research has shown that people with high scores for this trait tend to experience fewer stress symptoms and have better overall mental health [54,55]. In the nursing field, several studies have shown a direct association between hardiness and emotional exhaustion and an inverse relationship with quality of life [56,57], making it an essential trait for nurses in the acute care field [58], playing a protective role against stress and burnout. Our results support this association by suggesting that nurses with higher hardiness scores report lower prevalence and chronification of headaches. These data cannot be compared to the previous literature; as was the case for cognitive fusion, there has hardly been any focus in this regard in the context of the COVID-19 pandemic. A study conducted in HCWs in South Korea found that hardiness contributed significantly to post-traumatic growth after a highly stressful experience such as the COVID-19 pandemic [59]. Research in the general population during the COVID-19 pandemic has been more abundant and points to the protective role of hardiness in mental health [59], and specifically in anxiety, depression [21] and psychological well-being [60], suggesting that its negative association with intolerance to uncertainty might be a possible mechanism of action [61]. However, to the best of our knowledge, the relationship between hardiness and headaches has not been explored in any context. Therefore, the findings in the present study may be of special interest and should be the subject of further investigation in future research, insisting ultimately on the importance of psychosocial variables as risk and/or protective factors in headaches in ICU nurses. This fact has already been highlighted by different systematic reviews in relation to the mental health of HCWs, including issues such as post-traumatic stress, anxiety and depression [62,63], insisting on the need to go further and to clarify the psychological mechanisms involved.

The present study has certain limitations that should be taken into account. The sampling method was non-probabilistic of convenience, concentrating the population in certain communities and losing representativeness for the general population. Another limitation to be taken into account is that personality is a complex concept that includes multiple variables in addition to those contemplated in our study (cognitive fusion and hardiness). We would suggest that future lines of research take into consideration other personality variables (self-efficacy, tolerance to uncertainty, emotional regulation) and their influence on headaches. As an additional limitation, it would have been of interest to assess the previous headaches in the sample before the COVID-19 pandemic. The assessment of headache types would have been equally interesting. Another limitation is related to the lack of control of comorbidity associated with headache. On the one hand, as has been pointed out, the impact of stress on muscle tension changes and its influence on headaches should also addressed [7]. On the other hand, special mention should be made of temporomandibular disorders and bruxism, due to their higher prevalence in women [64] and their increase during the COVID-19 pandemic [64]. Given its close relationship with headaches [65], the evaluation of these disorders should be taken into account in future studies on this topic.

## 5. Conclusions

Despite the limitations of the present study, it is important to point out the main contributions of the findings, some of which are particularly novel in relation to the previous literature. Among them, the relevant role of the psychological variables related to symptoms (i.e., anxiety and burnout) and of the personality variables considered (i.e., hardiness and cognitive fusion) in the prevalence and maintenance of headaches in ICU nurses should be highlighted. Overall, the data indicate that it is important to address the psychological needs of nurses to prevent and manage headaches in the ICU work environment. Although multiple studies analyze the symptoms associated with the prevalence of headache in HCWs, there are very few studies that assess personality traits in their association with headache. Our study particularly highlights the intervention on hardiness and cognitive fusion.

## Figures and Tables

**Table 1:** Descriptive data of the sample and associations between headaches and different sociodemographic and occupational variables.

			Headaches at the First Time Point					Chronic Headaches				
	Mean (SD)	f (%)	Yes 93 (71%)	No 38 (29%)	Statistical Analysis			Yes 76 (58.8%)	No 54 (41.2%)	Statistical Analysis		
					*p* Value	χ2/t	Effect Size ^2^			*p* Value	χ2/t	Effect Size ^2^
Age	40.54 (10.02)		40.52 (9.41)	40.61 (11.52)	0.96	0.043	0.008	39.76 (9.47)	41.67 (10.76)	0.287	1.069	0.48
Work experience	11.76 (9.34)		11.45 (9.11)	12.50 (9.10)	0.56	0.581	0.11	11.18 (9.15)	12.57 (9.64)	0.404	0.838	0.14
**Gender**												
Male		15 (11.5)	7 (46.78%)	8 (53.3%)	0.03	4.868	3.27 [1.09,9.80]	7 (46.7%)	8 (53.3%)	0.311	1.026	1.73 [0.59,5.12]
Female		116 (88.5)	86 (74.1%)	30 (22.9%)				70 (60.3%)	46 (39.7%)			
**Family Status**												
Married/cohabiting		88 (67.2)	61 (69.3%)	27 (30.7%)	0.62	0.365	0.77 [0.34,1.76]	51 (58%)	37 (42%)	0.784	0.075	0.90 [0.42,1.89]
Single		43 (32.8)	32 (74.4%)	11 (25.9%)				26 (60.5%)	17 (39.5%)			
**Employment S** **^1^**												
Permanent		78 (59.5)	51 (65.4%)	27 (20.6%)	0.11	2.944	2.02 [0.89,4.55]	42 (53.8%)	36 (46.2%)	0.164	1.936	1.66 [0.81,3.43]
Not permanent		53 (40.5)	42 (79.2%)	11 (28.8%)				35 (66%)	18 (34.0%)			
**Transfer to ICU**												
Yes		29 (22.1)	23 (79.3%)	6 (20.7%)	0.26	1.251	1.75 [0.65,4.72]	17 (58.6%)	12 (41.4%)	0.984	0.001	0.99 [0.42,2.29]
No		102 (77.9)	70 (68.6%)	32 (31.4%)				60 (58.8%)	42 (41.2%)			
**PCR+**												
Yes		54 (41.2)	38 (70.4%)	16 (29.6%)	0.89	0.017	1.05 [0.49,2.26]	32 (59.3%)	22 (40.7%)	0.925	0.009	0.96 [0.47,1.96]
No		77 (58.8)	55 (71.4%)	22 (28.6%)				45 (58.4%)	32 (41.6%)			
**Availability of PPE**												
Yes or usually		68 (51.9)	45 (66.2%)	23 (33.8%)	0.20	1.592	0.61 [0.28,1.31]	38 (55.9%)	30 (44.1%)	0.484	0.490	0.77 [0.38,1.56]
No or rarely		63 (48.1)	48 (76.2%)	15 (23.8%)				39 (61.9%)	24 (38.1%)			

^1^ Employment status, ^2^ effect size: Cohen’s d for χ2, OR [95%CI] for Student’s *t*-test.

**Table 2:** Association between maintenance of headaches, symptoms and personality.

	Mean (SD)	Chronic Headaches		Statistical Analysis		
		Yes	No	*p* Value	t Student	Cohen’s d
Anxiety	8.64 (4.84)	9.64 (4.61)	7.22 (4.85)	**0.005**	−2.414	0.51
Insomnia	10.34 (6.01)	11.29 (6.14)	8.98 (5.60)	**0.030**	−2.304	0.39
Emotional Fatigue	26.98 (12.57)	29.08 (12.43)	23.98 (12.27)	**0.022**	−5.096	0.41
Depersonalization	6.30 (5.60)	6.86 (5.58)	5.50 (5.58)	0.173	−1.357	0.24
Reduced Personal Acc ^1^	13.16 (7.17)	14.16 (8.93)	11.77 (7.83)	0.117	−2.378	0.27
Cognitive Fusion	22.24 (10.29)	23.71 (10.20)	20.15 (23.71)	**0.049**	−3.566	0.19
Hardiness	66.53 (9.60)	66.01 (9.23)	68.69 (9.79)	**0.031**	3.672	0.28

^1^ Reduced personal accomplishment.

## Data Availability

Research data will be available upon request to the corresponding author.
